# Which is more important for cardiometabolic health: sedentary time, higher intensity physical activity or cardiorespiratory fitness? The Maastricht Study

**DOI:** 10.1007/s00125-018-4719-7

**Published:** 2018-09-10

**Authors:** Jeroen H. P. M. van der Velde, Nicolaas C. Schaper, Coen D. A. Stehouwer, Carla J. H. van der Kallen, Simone J. S. Sep, Miranda T. Schram, Ronald M. A. Henry, Pieter C. Dagnelie, Simone J. P. M. Eussen, Martien C. J. M. van Dongen, Hans H. C. M. Savelberg, Annemarie Koster

**Affiliations:** 1 Department of Nutrition and Movement Sciences, Maastricht University, P.O. Box 616, 6200 MD Maastricht, the Netherlands; 2 NUTRIM School for Nutrition and Translational Research in Metabolism, Maastricht University, Maastricht, the Netherlands; 3 Division of Endocrinology, Department of Internal Medicine, Maastricht University Medical Centre (MUMC+), Maastricht, the Netherlands; 4 CARIM School for Cardiovascular Diseases, Maastricht University, Maastricht, the Netherlands; 5 CAPHRI Care and Public Health Research Institute, Maastricht University, Maastricht, the Netherlands; 6 Department of Internal Medicine, Maastricht University Medical Centre (MUMC+), Maastricht, the Netherlands; 7 Heart and Vascular Centre, Maastricht University Medical Centre (MUMC+), Maastricht, the Netherlands; 8 Department of Epidemiology, Maastricht University, Maastricht, the Netherlands; 9 Department of Social Medicine, Maastricht University, Maastricht, the Netherlands

**Keywords:** Accelerometry, Physical activity, Physical fitness, Sedentary behaviour, The metabolic syndrome, Type 2 diabetes

## Abstract

**Aims/hypotheses:**

Our aim was to examine the independent and combined (cross-sectional) associations of sedentary time (ST), higher intensity physical activity (HPA) and cardiorespiratory fitness (CRF) with metabolic syndrome and diabetes status.

**Methods:**

In 1933 adults (aged 40–75 years) ST and HPA (surrogate measure for moderate to vigorous physical activity) were measured with the activPAL3. CRF was assessed by submaximal cycle–ergometer testing. Metabolic syndrome was defined according to the Adult Treatment Panel (ATP) III guidelines. Diabetes status (normal, prediabetes [i.e. impaired glucose tolerance and/or impaired fasting glucose] or type 2 diabetes) was determined from OGTT. (Multinomial) logistic regression analyses were used to calculate likelihood for the metabolic syndrome, prediabetes and type 2 diabetes according to ST, HPA and CRF separately and combinations of ST–CRF and HPA–CRF.

**Results:**

Higher ST, lower HPA and lower CRF were associated with greater odds for the metabolic syndrome and type 2 diabetes independently of each other. Compared with individuals with high CRF and high HPA (CRF_high_–HPA_high_), odds for the metabolic syndrome and type 2 diabetes were higher in groups with a lower CRF regardless of HPA. Individuals with low CRF and low HPA (CRF_low_–HPA_low_) had a particularly high odds for the metabolic syndrome (OR 5.73 [95% CI 3.84, 8.56]) and type 2 diabetes (OR 6.42 [95% CI 3.95, 10.45]). Similarly, compared with those with high CRF and low ST (CRF_high_–ST_low_), those with medium or low CRF had higher odds for the metabolic syndrome, prediabetes and type 2 diabetes, irrespective of ST. In those with high CRF, high ST was associated with significantly high odds for the metabolic syndrome (OR 2.93 [95% CI 1.72, 4.99]) and type 2 diabetes (OR 2.21 [95% CI 1.17, 4.17]). The highest odds for the metabolic syndrome and type 2 diabetes were observed in individuals with low CRF and high ST (CRF_low_–ST_high_) (OR [95% CI]: the metabolic syndrome, 9.22 [5.74, 14.80]; type 2 diabetes, 8.38 [4.83, 14.55]).

**Conclusions/interpretation:**

These data suggest that ST, HPA and CRF should all be targeted in order to optimally reduce the risk for the metabolic syndrome and type 2 diabetes.

**Electronic supplementary material:**

The online version of this article (10.1007/s00125-018-4719-7) contains peer-reviewed but unedited supplementary material, which is available to authorised users.

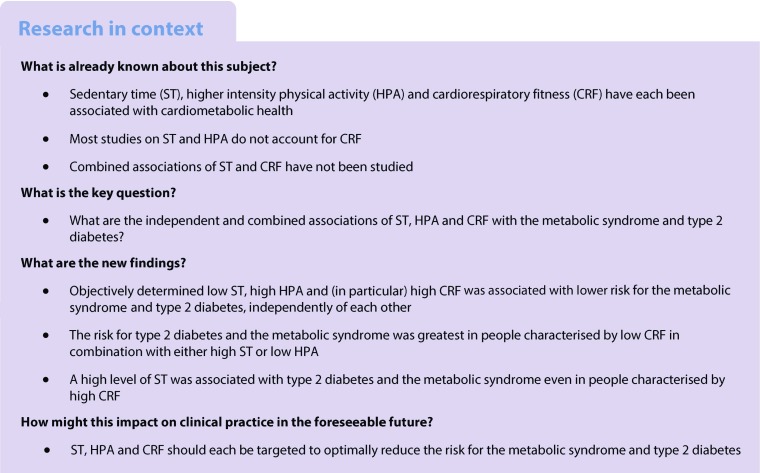



## Introduction

Globally, cardiovascular disease (CVD) is the leading cause of death and contributes substantially to accelerating healthcare costs [1]. Moderate to vigorous intensity physical activity (MVPA) is a key non-pharmacological strategy to reduce CVD risk [2]. However, MVPA comprises merely a small part of daily activities. Generally, the majority of the day is spent in sedentary behaviour [3]. Sedentary behaviour refers to any waking behaviour characterised by an energy expenditure ≤1.5 metabolic equivalents (METs) while seated or reclined [4]. Emerging evidence indicates that a large amount of sedentary time (ST) is a determinant of poor cardiometabolic health [5, 6]. Although this effect is probably independent of MVPA, the interrelationships between sedentary behaviour, MVPA and cardiometabolic health need further clarification, as recently concluded by the American Heart Association [7].

When examining the relationships between sedentary behaviour, MVPA and cardiometabolic health, cardiorespiratory fitness (CRF) should also be considered. CRF (or aerobic capacity) is an important determinant of cardiometabolic health [8, 9]. Differences in CRF between individuals are partly explained by differences in frequency and intensity of engagement in physical activity. Further, recent studies have shown an inverse association between ST and CRF [10–12]. Nonetheless, an estimated 10–50% of CRF is explained by factors other than physical activity, including genetic differences and behavioural or environmental elements [13]. Consequently, someone could engage regularly in MVPA and not have a high level of CRF, or could have a high level of CRF without frequently engaging in MVPA [14]. Thus, although MVPA, ST and CRF are interrelated to some extent, they should be considered different traits and may be independently associated with cardiometabolic health [14]. Studies that examined combined associations of CRF and physical activity reported clustering of the greatest cardiometabolic risk in people who had both low CRF and a low level of MVPA [15, 16]. Combined associations of CRF and ST have not previously been described.

Additional insight into sedentary behaviour, MVPA and CRF and their interrelationship as risk factors for cardiometabolic health may help to expand public health messages and policies aimed at preventing CVD. Therefore, the aim of this study was to examine the mutual independent and combined associations of ST, MVPA and CRF on cardiometabolic health and diabetes status.

## Methods

### Population

We used data from The Maastricht Study, an observational prospective population-based cohort study. The rationale and methodology have been described previously [17]. In brief, the study focuses on the aetiology, pathophysiology, complications and comorbidities of type 2 diabetes and is characterised by an extensive phenotyping approach. Individuals were eligible to participate in the study if they were aged between 40 and 75 years and living in the southern part of the Netherlands. Participants were recruited through mass media campaigns and from the municipal registries and the regional Diabetes Patient Registry via regular mail. Recruitment was stratified according to known type 2 diabetes status, with an oversampling of individuals with type 2 diabetes, for reasons of efficiency. The present report includes cross-sectional data from a selection of the first 3451 participants, who completed the baseline survey between November 2010 and September 2013. The study complies with the Declaration of Helsinki and has been approved by the institutional medical ethical committee (NL31329.068.10) and the Minister of Health, Welfare and Sports of the Netherlands (Permit 131088-105234-PG). All participants gave written informed consent.

### Cardiometabolic outcomes

The metabolic syndrome and diabetes status were used as main outcomes. In addition, the following individual outcome measures were used: waist circumference, blood pressure, plasma levels of HDL-cholesterol, triacylglycerol and glucose and homeostatic model assessment insulin resistance (HOMA2-IR). Details of assessment have been described previously [17]. The metabolic syndrome was defined according to the Adult Treatment Panel (ATP) III guidelines [18]. To determine diabetes status according to the WHO 2006 criteria [19], all participants (except those who used insulin) underwent an OGTT after an overnight fast as described elsewhere [17]. Participants were categorised as having normal glucose, prediabetes (impaired fasting glucose and/or impaired glucose tolerance; fasting plasma glucose 6.1–6.9 mmol/l and/or 2 h plasma glucose ≥7.8 to- &lt;11.1 mmol/l), or type 2 diabetes (fasting plasma glucose ≥7.0 mmol/l and/or 2 h plasma glucose ≥11.1 mmol/l). Participants taking glucose-lowering medication were also considered as having type 2 diabetes. Medication use was assessed during a medication interview where generic name, dose and frequency were registered. HOMA2-IR was calculated using the HOMA calculator, available from https://www.dtu.ox.ac.uk (accessed 31 March 2016).

### Accelerometry

Daily activity levels were measured using the activPAL3 physical activity monitor (PAL Technologies, Glasgow, UK). The activPAL3 is a small (53 × 35 × 7 mm), lightweight (15 g) triaxial accelerometer that determines posture (sitting/lying, standing, stepping) based on acceleration information. Participants were asked to wear the accelerometer on the right thigh for 8 consecutive days without removing it at any time. Data were uploaded using the activPAL software and processed using customised software written in MATLAB R2013b (MathWorks, Natick, MA, USA). Data from the first day were excluded from the analysis. In addition, data from the final wear day providing ≤14 wear h of data were excluded from the analysis. Participants were included if they provided at least one valid day (≥10 h of waking data).

ST was calculated as the mean time spent in a sedentary position during waking time per day. The total amount of physical activity was calculated as the mean time stepping during waking time per day. Further, physical activity (stepping time) was classified as higher intensity physical activity (HPA) when step frequency &gt;110 steps/min, and was used as a proxy for MVPA [20]. Details and the method used to determine waking time have been described previously [21].

### CRF

As a measure of CRF, estimated maximum power output (W_max_) adjusted for body weight (W_max_ kg^−1^) was used. W_max_ was estimated from a graded submaximal exercise protocol performed on a cycle–ergometer system (CASETM version 6.6 in combination with e-bike; GE Healthcare, Milwaukee, WI, USA). For safety reasons, participants with recent or manifest cardiovascular complications were excluded from the exercise test. The protocol has been described in detail elsewhere [12]. W_max_ kg^−1^ was transformed into oxygen consumption ($\overset{\cdot }{V}{\mathrm{O}}_{2\mathrm{max}}$) using the following formula from the American College of Sports Medicine [22]: $\overset{\cdot }{V}{\mathrm{O}}_{2\mathrm{max}}$(ml kg^−1^ min^−1^) = (10.8 × W_max_ kg^−1^) + 7.

### Covariates

Questionnaires were used to collect information on age (in years), sex, educational level (highest completed education, subsequently classified as low, middle and high), smoking behaviour (non-smoker, former smoker and current smoker), alcohol consumption (non-consumer, low-consumer [women ≤7 glasses per week, men ≤14 glasses per week] and high-consumer [women &gt;7 glasses per week, men &gt;14 glasses per week]), CVD history (derived from the Rose questionnaire and defined as a self-reported history of any of the following conditions: myocardial infarction, cerebrovascular infarction or haemorrhage and percutaneous artery angioplasty of, or vascular surgery on, the coronary, abdominal, peripheral or carotid arteries) [23], mobility limitations (defined as having difficulty walking 500 m or climbing stairs) and energy intake (calculated as the mean energy intake per day from a frequent food questionnaire). Percentage of body fat was calculated with the Siri equation [24] after estimating body density from skinfold thickness at four sites (suprailiac, subscapula, biceps and triceps) according to Durnin and Womersley [25].

### Statistical analyses

First, population characteristics were provided as means (± SD), median (25–75%) or percentages as appropriate.

Second, (multinomial) logistic regression analyses were performed for the outcomes metabolic syndrome and diabetes status. Associations in model 1 were adjusted for age, sex, waking time, education level, smoking status, alcohol consumption, mobility limitation, CVD history and energy intake. Glucose metabolism was additionally adjusted for body fat percentage. Associations in models 2a, 2b and 2c were additionally adjusted for ST, HPA and CRF, respectively. To examine their relative importance in cardiometabolic outcomes, ST, HPA and CRF were expressed per 1 SD.

Third, combined associations of ST–CRF and HPA–CRF with the metabolic syndrome and diabetes status were analysed. For this, CRF was categorised into tertiles (CRF_low_, CRF_medium_ and CRF_high_) based on sex and age (40–49, 50–59, 60–69 and &gt;70 years). CRF values for each age- and sex-specific tertiles are provided in electronic supplemental material (ESM) Table 1. Further, proportions of daily ST and HPA were categorised into sex-specific tertiles (ST_high_ ST_medium_ and ST_low_ and HPA_low_, HPA_medium_, and HPA_high_, respectively). For men, tertile cut points were 59% and 67% for ST and 1.0% and 2.3% for HPA. For women, tertile cut points were 52% and 60% for ST and 1.9% and 3.3% for HPA. Tertiles of CRF and HPA and tertiles of CRF and ST were combined into nine subgroups. For each subgroup, the odds for the metabolic syndrome and prediabetes and type 2 diabetes were calculated. These analyses were adjusted for the same covariates as described in model 1 above.

Fourth, in additional analyses, linear regression analyses were performed to assess the independent association of ST, HPA and CRF with individual cardiometabolic outcome measures. Adjustments were similar to those for model 1 (described above), with the addition of antihypertensive and lipid-modifying medication use.

Fifth, the combined effects of ST–CRF and HPA–CRF on individual markers of cardiometabolic health were examined by calculating adjusted means for all subgroups of ST–CRF and HPA–CRF using general linear models. The adjusted means of subgroups based on CRF and HPA were additionally adjusted for ST. The adjusted means of subgroups based on CRF and ST were additionally adjusted for HPA.

In all analyses men and women were analysed together, as no interaction effect of sex was observed. In sensitivity analyses, all analyses were repeated after excluding participants with mobility limitations (*n* = 341).

## Results

Population characteristics are described in Table 1. Data were available for 1993 participants after excluding those who did not receive an accelerometer due to logistics (*n* = 673), those with invalid accelerometer readings (*n* = 136), those without (*n* = 282) or with invalid (*n* = 144) CRF measurement, those with missing data on cardiometabolic outcome variables (*n* = 8), those with missing covariates (*n* = 195) and those with type 1 diabetes or other forms of diabetes, including latent autoimmune diabetes of adults (LADA), steroid-induced diabetes and diabetes after pancreatectomy (*n* = 20).

**Table 1:** Characteristics of the study population (*N* = 1993)

Characteristic	Value
Age, years	59.7 (8.1)
Sex, % male	49.4
Educational level, % high	39.7
Smoking status, % current	12.0
Alcohol consumption, % high	25.3
Mobility limitation, %	17.1
History of CVD, % yes	13.9
BMI, kg/m^2^	26.7 (4.3)
Body fat percentage	33.9 (7.0)
Waist circumference, cm	94.6 (13.1)
Systolic BP, mmHg	134.5 (17.7)
Diastolic BP, mmHg	76.2 (9.8)
Plasma triacylglycerol, mmol/l	1.2 (0.9–1.7)
Plasma HDL-cholesterol, mmol/l	1.6 (0.5)
Fasting plasma glucose, mmol/l	5.5 (5.0–6.4)
HOMA-IR^a^	1.4 (1.0–2.1)
Medication, %	
Glucose-lowering	20.1
Antihypertensive	37.1
Lipid-lowering	34.4
Metabolic syndrome, %	36.0
Glucose metabolism status, %	
Normal	58.9
Prediabetes	15.7
Type 2 diabetes	25.4
Valid days of accelerometer wear, *n*	6.3 (1.2)
Waking time, h/day	15.7 (0.9)
ST, h/day	9.3 (1.6)
Total physical activity, h/day	2.0 (0.7)
HPA, min/day	19.5 (9.9–32.0)
CRF	
W_max_, W kg^−1^	2.1 (0.6)
$\overset{\cdot }{V}{\mathrm{O}}_{2\mathrm{max}}$, ml min^−1^ kg^−1^	30.1 (6.2)

Values are expressed as mean (SD), median (25–75%) or percentages

^a^*N* = 1893

Table 2 provides details of the independent associations between ST, HPA and CRF and the likelihood of the metabolic syndrome, prediabetes and type 2 diabetes. Longer ST was associated with greater odds for the metabolic syndrome and type 2 diabetes, independent of HPA and CRF (models 2b and 2c). More time engaged in HPA was associated with lower odds for the metabolic syndrome independent of ST and CRF. In addition, HPA was associated with lower odds for type 2 diabetes independent of ST (model 2a) but not independent of CRF (model 2c). Higher CRF was associated with lower odds for the metabolic syndrome, prediabetes and type 2 diabetes independent of ST and HPA (models 2a and 2b).

**Table 2:** OR for metabolic syndrome, prediabetes and type 2 diabetes per 1 SD difference in ST, HPA and CRF

Activity/fitness	Model 1	Model 2a (Model 1+ST)	Model 2b (Model 1+HPA)	Model 2c (Model 1+CRF)
ST^a^				
Metabolic syndrome	1.57 (1.4, 1.76)		1.42 (1.26, 1.59)	1.35 (1.19, 1.52)
Prediabetes	1.13 (0.98, 1.30)		1.11 (0.96, 1.28)	1.10 (0.95, 1.27)
Type 2 diabetes	1.43 (1.25, 1.63)		1.35 (1.18, 1.55)	1.32 (1.15, 1.51)
HPA^b^				
Metabolic syndrome	0.61 (0.53, 0.69)	0.67 (0.59, 0.76)		0.78 (0.68, 0.89)
Prediabetes	0.92 (0.80, 1.06)	0.94 (0.81, 1.09)		0.98 (0.85, 1.14)
Type 2 diabetes	0.69 (0.60, 0.81)	0.75 (0.64, 0.87)		0.85 (0.73, 1.00)
CRF^c^				
Metabolic syndrome	0.38 (0.33, 0.43)	0.40 (0.35, 0.46)	0.42 (0.36, 0.49)	
Prediabetes	0.73 (0.62, 0.87)	0.74 (0.62, 0.89)	0.74 (0.62, 0.88)	
Type 2 diabetes	0.43 (0.36, 0.51)	0.46 (0.38, 0.55)	0.45 (0.38, 0.54)	

Associations, expressed as OR (95% CI), in model 1 were adjusted for age, sex, waking time, education, mobility limitation, smoking status, alcohol consumption, (history of) CVD and energy intake. The models for prediabetes and type 2 diabetes were additionally adjusted for fat percentage

^a^Each unit change (1 SD) corresponds to 1.63 h for ST

^b^Each unit change (1 SD) corresponds to 18.22 min for HPA

^c^Each unit change (1 SD) corresponds to 0.58 W_max_ kg^−1^ (or 6.23 ml min^−1^ kg^−1^) for CRF

**Fig. 1:**
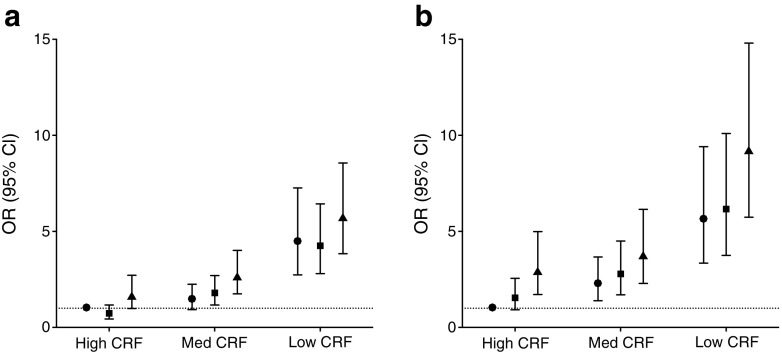
Associations with the metabolic syndrome in subgroups combined from HPA and CRF (**a**) and combined from ST and CRF (**b**). Associations were adjusted for age, education level, smoking status, alcohol consumption, mobility limitation, (history of) CVD and energy intake. In addition, subgroups based on CRF and HPA were adjusted for ST and vice versa. The smallest subgroups were CRF_high_–HPA_low_ and CRF_low_–HPA_high_ (both *n* = 118) and the largest subgroups were CRF_low_–HPA_low_ (*n* = 330) and CRF_high_–HPA_high_ (*n* = 328). Circles, high HPA (**a**) or low ST (**b**); squares, medium HPA (**a**) or medium ST (**b**); triangles, low HPA (**a**) or high ST (**b**). Med, medium

Figure 1a shows the associations of the combined tertiles of CRF and HPA with the metabolic syndrome. Compared with people with CRF_high_–HPA_high_ (reference group), the subgroups with medium or low CRF had higher odds for the metabolic syndrome, with the greatest odds in the CRF_low_–HPA_low_ subgroup (OR 5.73 [3.84, 8.56]). Odds for the metabolic syndrome were greater in people with CRF_low_–HPA_high_ (OR 4.46 [2.74, 7.26]) than in those with CRF_high_–HPA_low_ (OR 1.64 [0.99, 2.72]). When analysing the contribution of ST (Fig. 1b), people with higher ST and with medium or low CRF had greater odds for the metabolic syndrome compared with those with CRF_high_–ST_low_ (reference group). The highest odds for the metabolic syndrome were seen in those with CRF_low_–ST_high_ (OR 9.22 [5.74, 14.80]). In addition, people with CRF_high_–ST_high_ had greater odds for the metabolic syndrome (OR 2.93 [1.72, 4.99]) than those in the reference group. Further, the odds for the metabolic syndrome in the CRF_low_–ST_low_ subgroup (OR 5.62 [3.35, 9.41]) were greater than the odds in the CRF_high_–ST_high_ subgroup (OR 2.93 [1.72, 4.99]).

**Fig. 2:**
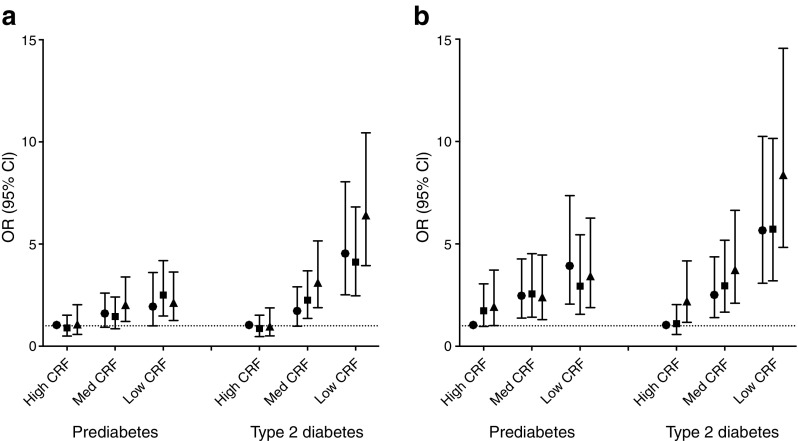
Associations with diabetes status (prediabetes and type 2 diabetes) in subgroups combined from HPA and CRF (**a**) and combined from ST and CRF (**b**). Associations were adjusted for age, education level, smoking status, alcohol consumption, mobility limitation, (history of) CVD, energy intake and fat percentage. In addition, subgroups based on CRF and HPA were adjusted for ST and vice versa. The smallest subgroups were CRF_high_–ST_high_ (*n* = 153) and CRF_low_–ST_low_ (*n* = 154), largest subgroups were CRF_low_–ST_high_ (*n* = 311) and CRF_high_–ST_low_ (*n* = 271). Circles, high HPA (**a**) or low ST (**b**); squares, medium HPA (**a**) or medium ST (**b**); triangles, low HPA (**a**) or high ST (**b**). Med, medium

Figure 2a shows the associations of the combined tertiles of CRF and HPA with diabetes status. Compared with the CRF_high_–HPA_high_ subgroup (reference group), the odds for prediabetes were higher in people with low CRF and CRF_medium_–HPA_low_. The odds for type 2 diabetes were greater in those with medium and low CRF (with the exception of CRF_medium_–HPA_high_) when compared with the reference group, with greatest odds occurring in the CRF_low_–HPA_low_ group (OR 6.42 [3.95, 10.45]). In participants with high CRF, all levels of HPA had similar odds for type 2 diabetes. Figure 2b shows the associations of the combined tertiles of CRF and ST with diabetes status. Compared with people in the CRF_high_–ST_low_ subgroup (reference group), the odds for prediabetes were greater in all low and medium CRF subgroups and in the subgroup CRF_high_–ST_high_. Using the same reference group for comparison, all low and medium CRF subgroups had greater odds for type 2 diabetes. Further, people with CRF_high_–ST_high_ had increased odds for type 2 diabetes as well (OR 2.21 [1.17, 4.17]) but this was lower than the odds for people with CRF_low_–ST_low_ (OR 5.62 [3.35, 9.41]). The highest OR for type 2 diabetes was seen in the CRF_low_–ST_high_ subgroup: OR 8.38 (4.83, 14.55).

ESM Table 2 shows the mutually independent associations of ST, HPA and CRF with individual markers of cardiometabolic health. More ST was associated with larger waist circumference, lower HDL-cholesterol, higher triacylglycerol and higher fasting glucose levels and higher HOMA2-IR, independent of HPA and CRF. Greater HPA was associated with smaller waist circumference, higher HDL-cholesterol and lower triacylglycerol levels and lower HOMA2-IR, independent of ST and CRF. Higher CRF was associated with smaller waist circumference, lower diastolic blood pressure, higher HDL-cholesterol, lower triacylglycerol and lower glucose levels and lower HOMA2-IR, independent of HPA and ST.

ESM Figs 1 and 2 show the associations of combined tertiles of CRF and HPA, and CRF and ST, with individual markers of cardiometabolic health. The largest differences in adjusted means of cardiometabolic markers were between low and high CRF. In addition, even at high CRF, lower levels of HPA and greater ST were associated with lower levels of HDL-cholesterol and higher triacylglycerol.

In additional analyses, all participants with mobility limitations (*n* = 341) were excluded from analyses. This did not noticeably affect any of the associations presented (data not tabulated).

## Discussion

Several studies have highlighted the importance of ST, physical activity and CRF for cardiometabolic health individually. However, when developing preventive strategies these factors should not be viewed in isolation, as they occur concurrently. This cross-sectional study is, to our knowledge, the first to examine the independent and combined associations of ST, HPA and CRF with cardiometabolic health.

Our results show that ST, independent of HPA and CRF, is associated with poor cardiometabolic health and type 2 diabetes. Associations between greater ST and poor cardiometabolic health, independent of HPA, have been described previously [5, 6]. Recently, we reported that in The Maastricht Study cohort each extra hour of ST was associated with a 22% increased odds for type 2 diabetes and a 39% increased odds for the metabolic syndrome [26]. Associations between objectively measured ST and poor cardiometabolic health, independent of CRF, have been reported in some [27, 28], but not all, earlier studies [29–31]. From the combined associations of CRF–ST, we showed that, overall, harmful outcomes associated with low ST vs high ST were outweighed by the harmful outcomes associated with having a lower level of CRF. Further, the odds for the metabolic syndrome and type 2 diabetes were also greater in the CRF_high_–ST_high_ subgroup, indicating that even individuals with high CRF may be at increased risk for metabolic diseases due to prolonged sitting.

In line with our results, others have reported beneficial associations of HPA with cardiometabolic risk, independent of CRF [15, 28, 32–37]. Although CRF has been reported to have a large effect size when comparing associations of HPA and CRF with health, [28, 32, 35, 36], studies on the combined associations of HPA–CRF with cardiometabolic outcomes are scarce. One study observed that individuals with CRF_high_–HPA_low_ were characterised by a healthier cardiometabolic risk profile than those with CRF_low_–HPA_high_ [15]. A prospective study examining the incidence of type 2 diabetes reported similar results [37]. This seems to agree with our results: compared with CRF_high_–HPA_high_, all HPA subgroups with low CRF had higher odds for the metabolic syndrome, prediabetes and type 2 diabetes, regardless of the level of HPA. A high level of HPA was sufficient to ‘counteract’ the detrimental associations in subgroups with medium CRF only and did not seem to have additional benefit in people with high CRF.

The relative importance of HPA and ST vs CRF for cardiometabolic health should be discussed in light of the mediating effects of CRF. Mediation analyses have shown that CRF explained 73% [38], or more [35], of the associations between MVPA and metabolic risk. Biological pathways through which HPA affects cardiometabolic health could therefore be similar to those for CRF. In addition, recent studies have observed an association between high ST and lower CRF [10, 11], implying that the association between ST and cardiometabolic health could also be partly mediated through lower CRF [38]. Nonetheless, results from our joint analyses showed an elevated risk for the metabolic syndrome and type 2 diabetes in the CRF_high_–ST_high_ subgroup, suggesting that other mechanisms are involved as well. Subsequently, high CRF may not be sufficient to ‘counteract’ the deleterious health outcomes associated with sedentary behaviour.

Crossover studies in sedentary individuals with and without type 2 diabetes suggest that sedentary behaviour has negative cardiometabolic effects that are independent of changes in energy balance. Replacing ST with regular short bouts of light intensity physical activity (which presumably has a relatively small effect on CRF) had more positive cardiometabolic effects than HPA in some studies [39, 40]. Reduced activity of AMP-activated protein kinase (AMPK) and lipoprotein lipase (LPL) activity, due to contractile inactivity of skeletal muscles, could be important underlying mechanisms for the effects of prolonged ST on glucose and lipid metabolism [41]. Physical activity is usually associated with increased blood-flow-induced shear stress on the vascular endothelium, which plays an important role in maintaining vascular homeostasis. Endothelial dysfunction, a key event in the development of CVD, could therefore be another consequence of prolonged ST [42]. Since research into sedentary behaviour is a relatively young field, studies investigating biological mechanisms explaining the detrimental effects of prolonged ST are warranted.

Future work should also focus on dose–response: how much ST is associated with a clinically relevant increase in risk and what levels of HPA and CRF are associated with a clinically relevant lower risk for the metabolic syndrome and type 2 diabetes? In this study, low, medium and high levels of CRF, ST and HPA were derived from data-driven tertiles. Thus, the cut points between tertiles may not represent clinically relevant cut-off points. Globally, the daily amount of ST is increasing while the amount of MVPA is decreasing [3]. Presently, people generally spend the majority of the day in sedentary behaviour. Thus, although the strength of the associations of ST with cardiometabolic health was relatively small compared with that of CRF, reducing ST potentially has a great impact on public health due to its high prevalence. Whether sitting time should be reduced by increasing the daily amount of light intensity physical activity or whether relatively brief periods of MVPA are sufficient to improve cardiometabolic risk is still debated [7].

The strengths of this study include the use of a posture-based activity monitor to assess ST and time spent in HPA. However, our results should also be interpreted in the light of some limitations. Importantly, causality should be interpreted with caution due to the cross-sectional nature of this study. For instance, people may change their behaviour due to illness. However, we attempted to eliminate these influences by adjusting our analyses for mobility limitations. Moreover, repeating the analyses after excluding those with mobility limitations did not alter our findings. Further, HPA was based on step frequency, which may be a less precise method of determining the intensity of physical activity compared with methods based on accelerometry data. However, the applied frequency of &gt;110 steps/min has been reported to correspond to a MET score of &gt;3.0 [20]. Therefore, it may be interpreted as an approximation of MVPA. In addition, selection bias may have been introduced due to exclusion criteria applied for the submaximal exercise test. Consequently, participants with recent cardiovascular complications have been excluded, possibly resulting in a slight underestimation of true effect sizes (since the study population is healthier than the general population). Finally, The Maastricht Study comprises mainly individuals of European descent, including participants with type 2 diabetes who have well-controlled blood glucose. This limits its generalisability to other populations.

In conclusion, high ST, low HPA and low CRF were each associated with several markers of cardiometabolic health and with higher risk for the metabolic syndrome and type 2 diabetes, independently of each other. The combinations CRF_low_–HPA_low_ and CRF_low_–ST_high_ were associated with a particularly high risk of developing the metabolic syndrome and type 2 diabetes. A shift from low to medium CRF was associated with greatest reduction in risk for having the metabolic syndrome and type 2 diabetes. Additionally, reducing ST as well as increasing HPA was associated with additive risk reductions and in relative terms the strengths of these associations were comparable. To improve cardiovascular risk and to prevent type 2 diabetes, these data support the development of new strategies that target all three components—ST, HPA and CRF.

## Electronic supplementary material

ESM (PDF 560 kb)

## Data Availability

Data are unsuitable for public deposition due to ethical restriction and privacy of participant data according to the approved study protocol by the institutional medical ethical committee (Medisch-ethische toetsingscommissie azM/UM, NL31329.068.10) and the Minister of Health, Welfare and Sports of the Netherlands (Permit 131088-105234-PG). Data are available from The Maastricht Study for any interested researcher who meets the criteria for access to confidential data. The Maastricht Study Management Team (research.dms@mumc.nl) and the last author (a.koster@maastrichtuniversity.nl) may be contacted to request data.

## Abbreviations

CRF: Cardiorespiratory fitness
CVD: Cardiovascular disease
HPA: Higher intensity physical activity
MET: Metabolic equivalent
MVPA: Moderate to vigorous physical activity
$\overset{\cdot }{V}{\mathrm{O}}_{2\mathrm{max}}$: Maximum rate of oxygen consumption
ST: Sedentary time
W_max_: Maximum power output
