# Supplementary material for: Which is more important for cardiometabolic health: sedentary time, higher intensity physical activity or cardiorespiratory fitness? The Maastricht Study
Source: Diabetologia. 2018 Sep 10;61(12):2561–9. doi: 10.1007/s00125-018-4719-7 (PMC6223836; doi:10.1007/s00125-018-4719-7)
Supplement: Supplementary file 1 — (PDF 560 kb) [file 125_2018_4719_MOESM1_ESM.pdf]

ESM table 1: Range of VO_{2max} (ml min^{-1} kg^{-1}) in tertiles (low, medium, high) of CRF per sex and age group

		**Low CRF**		**Medium CRF**		**High CRF**	
		Min	Max	Min	Max	Min	Max
**Men**	40-49 yr	18.36	30.64	30.77	37.54	37.64	54.70
	50-59 yr	14.41	30.32	30.48	35.00	35.33	50.97
	60-69 yr	11.23	28.32	28.33	33.38	33.44	51.58
	>70 yr	15.46	24.63	24.66	28.84	28.92	44.46
**Women**	40-49 yr	17.47	29.03	29.22	34.32	34.34	47.77
	50-59 yr	13.17	27.64	27.77	32.18	32.29	46.32
	60-69 yr	15.13	25.53	25.53	29.61	29.65	47.18
	>70 yr	13.53	22.22	22.43	25.88	25.94	36.72

ESM table 2: Differences in markers of cardio metabolic health per one standard deviation difference in sedentary time, higher intensity physical activity, and cardio-respiratory fitness

		Model 1		Model 2a: Model 1 + ST		Model 2b: Model 1 + HPA		Model 2c: Model 1 + CRF	
		B	*95% CI*	*B*	*95% CI*	*B*	*95% CI*	*B*	*95% CI*
**Sedentary time**	Waist circumference (cm)	**2.15**	**(1.65; 2.64)**			**1.64**	**(1.13; 2.15)**	**1.39**	**(0.93; 1.85)**
	Systolic BP (mmHg)	-0.45	(-1.25; 0.34)			-0.51	(-1.33; 0.31)	-0.54	(-1.34; 0.26)
	Diastolic BP (mmHg)	0.41	(-0.05; 0.87)			0.37	(-0.10; 0.84)	0.34	(-0.12; 0.80)
	HDL-cholesterol (mmol/l)	**-0.05**	**(-0.07; -0.03)**			**-0.04**	**(-0.06; -0.02)**	**-0.05**	**(-0.07; -0.03)**
	Triacylglycerol (mmol/l)*	**1.06**	**(1.04; 1.09)**			**1.05**	**(1.02; 1.07)**	**1.05**	**(1.03; 1.07)**
	Fasting glucose (mmol/l)*	1.01	(1.00; 1.01)			1.01	(1.00; 1.01)	1.01	(1.00; 1.01)
	HOMA2-IR *	**1.05**	**(1.02; 1.07)**			**1.04**	**(1.02; 1.07)**	**1.04**	**(1.02; 1.07)**
**Higher intensity physical activity**	Waist circumference (cm)	**-2.21**	**(-2.68; -1.73)**	**-1.78**	**(-2.26; -1.29)**			**-1.01**	**(-1.47; -0.55)**
	Systolic BP (mmHg)	-0.10	(-0.85; 0.66)	-0.22	(-1.00; 0.56)			0.06	(-0.72; 0.84)
	Diastolic BP (mmHg)	-0.23	(-0.66; 0.21)	-0.14	(-0.59; 0.31)			-0.09	(-0.54; 0.37)
	HDL-cholesterol (mmol/l)	**0.07**	**(0.05; 0.08)**	**0.06**	**(0.04; 0.08)**			**0.06**	**(0.04; 0.08)**
	Triacylglycerol (mmol/l)*	**0.96**	**(0.93; 0.98)**	**0.96**	**(0.94; 0.98)**			**0.96**	**(0.94; 0.98)**
	Fasting glucose (mmol/l)*	1.00	(0.99; 1.01)	1.00	(0.99; 1.01)			1.00	(0.99; 1.01)
	HOMA2-IR *	**0.96**	**(0.94; 0.99)**	**0.97**	**(0.95; 1.00)**			**0.97**	**(0.95; 1.00)**
**Cardio-respiratory fitness**	Waist circumference (cm)	**-5.15**	**(-5.65; -4.64)**	**-4.89**	**(-5.39; 4.38)**	**-4.81**	**(-5.34; 4.29)**		
	Systolic BP (mmHg)	-0.72	(-1.64; 0.20)	-0.80	(-1.72; 0.13)	-0.74	(-1.69; 0.21)		
	Diastolic BP (mmHg)	**-0.70**	**(-1.23; -0.17)**	**-0.65**	**(-1.18; -0.12)**	**-0.67**	**(-1.22; -0.12)**		
	HDL-cholesterol (mmol/l)	**0.05**	**(0.02; 0.07)**	**0.04**	**(0.02; 0.06)**	**0.03**	**(0.00; 0.05)**		
	Triacylglycerol (mmol/l)*	**0.96**	**(0.93; 0.98)**	**0.96**	**(0.94; 0.99)**	**0.97**	**(0.94; 1.00)**		
	Fasting glucose (mmol/l)*	**0.99**	**(0.98; 1.00)**	**0.99**	**(0.98; 1.00)**	**0.99**	**(0.98; 1.00)**		
	HOMA2-IR *	**0.95**	**(0.92; 0.97)**	**0.95**	**(0.93; 0.98)**	**0.96**	**(0.93; 0.98)**		

HOMA2-IR, homeostatic model assessment insulin resistance; BP, blood pressure. Associations (expressed as regression coefficients (B) with 95% CI) in model 1 adjusted for: age, sex, waking time, education, mobility limitation, fat percentage (with exception of outcome waist circumference), antihypertensive and lipid modifying medication, diabetes status (with exception of the outcome fasting glucose and HOMA-IR, that were adjusted for glucose lowering medication), smoking status, alcohol consumption, (history of) cardiovascular diseases and energy intake. *back-transformed from log-scale and should therefore be interpreted as proportional change (B <0 indicates negative association, B = 1 indicates no association and B >1 indicates a positive association). Each unit change (i.e. 1 SD) corresponds to 1.63 hours for ST, 18.22 minutes for HPA, and 0.58 W kg^{-1} (or 6.21 ml min^{-1} kg^{-1}) for CRF. (HOMA2-IR was available for N=1,883 due to missing insulin values)

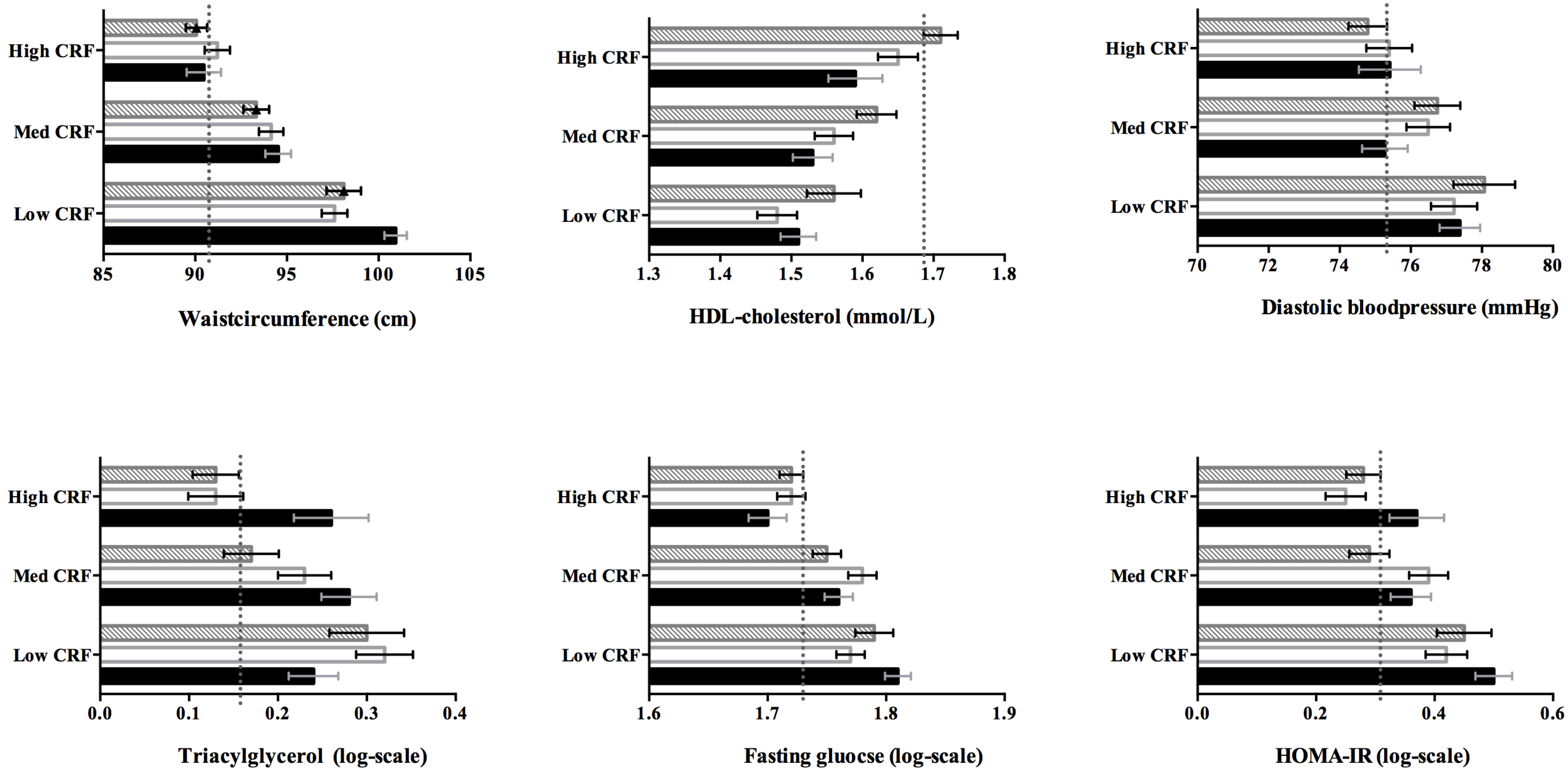

ESM Figure 1: Markers of cardio metabolic health (adjusted means with SE) in subgroups combined from higher intensity physical activity (HPA) and CRF. Associations were adjusted for age, education level, smoking status, alcohol consumption, percentage of body fat (except waist circumference), diabetes status (except fasting glucose and HOMA-IR), anti-hypertensive and lipid-modifying medication, mobility limitation, (history of) CVD, energy intake, and ST. Striped bars indicate high HPA, white bars medium HPA, and black bars low HPA.

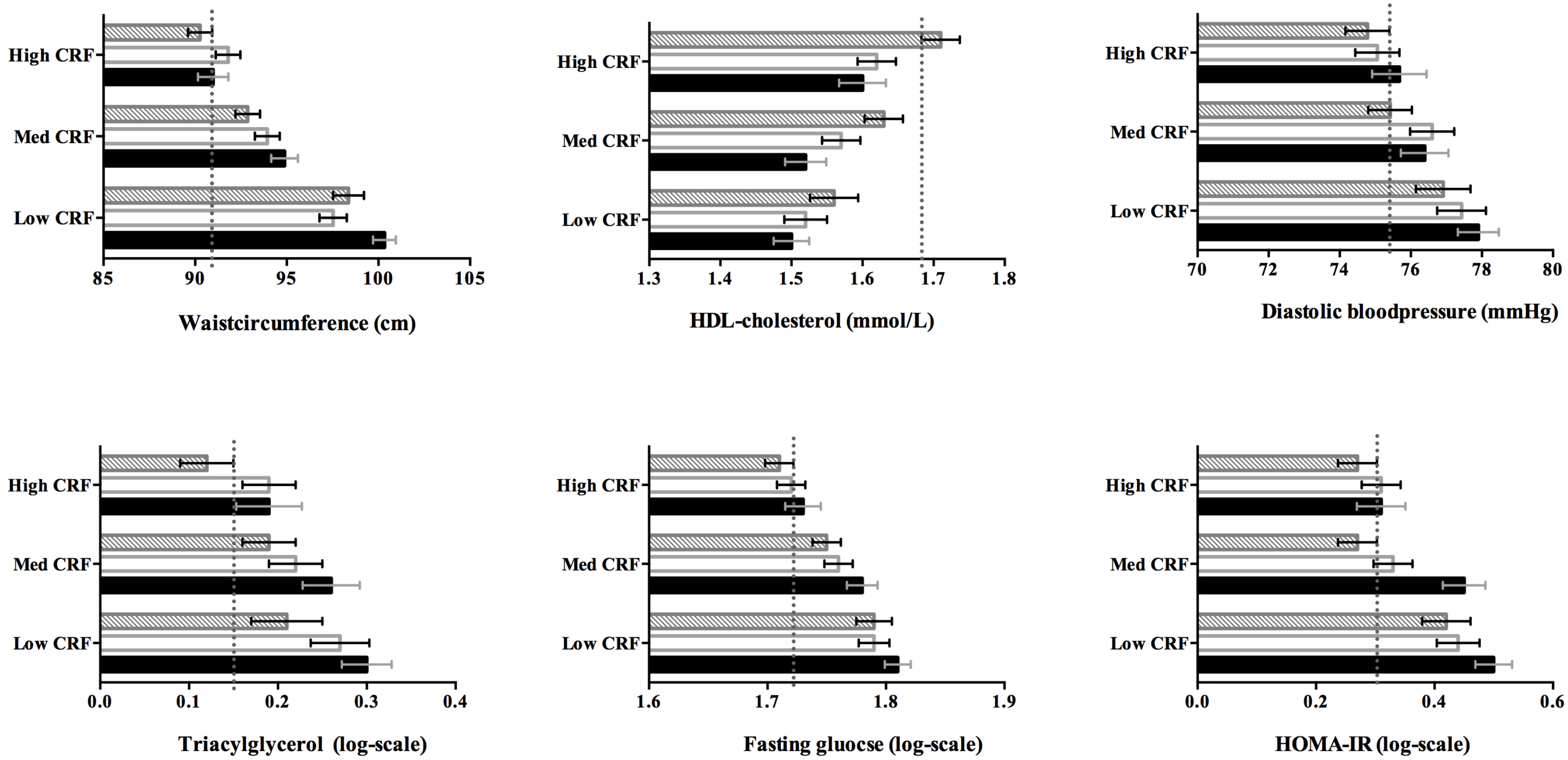

ESM Figure 2: Markers of cardio metabolic health (adjusted means with SE) in subgroups combined from sedentary time (ST) and CRF. Associations were adjusted for age, education level, smoking status, alcohol consumption, percentage of body fat (except waist circumference), diabetes status (except fasting glucose and HOMA-IR), anti-hypertensive and lipid-modifying medication, mobility limitation, (history of) CVD, energy intake, and HPA. Striped bars indicate low ST, white bars medium ST, and black bars high ST.
